# Short report: knowledge and perceptions of health workers that strengthen adherence for paediatric and adolescent clients on the intensive adherence counselling program in Kampala, Uganda: a qualitative study

**DOI:** 10.4314/ahs.v21i1.5S

**Published:** 2021-05

**Authors:** Esther Nasuuna, Joanita Kigozi, Alex Muganzi, Nelson Sewankambo, Damalie Nakanjako

**Affiliations:** 1 Infectious Diseases Institute, Makerere University, Kampala, Uganda; 2 Department of Medicine, School of Medicine, College of Health Sciences, Makerere University, Kampala Uganda

**Keywords:** Intensive adherence counselling, health care worker barriers

## Abstract

**Background:**

Health care workers (HWs) support HIV positive children and adolescents with detectable HIV viral loads on the intensive adherence counselling (IAC) program to achieve viral suppression through individual adherence counselling. Low re-suppression rates of 23% showed low program effectiveness in fifteen public health facilities.

**Objectives:**

We set out to determine the knowledge and perceptions of HWs that support this program to improve its effectiveness.

**Methods:**

We conducted a qualitative study where five HWs that oversee clinical care for children on ART were interviewed about the program. Data on their knowledge of the program, and perceptions on why it was not effective was collected. Thematic analysis using the inductive approach was used. Transcripts were read, coded and emergent themes determined.

**Results:**

Five HWs participated and all were knowledgeable about the program. Two themes emerged as barriers to IAC program effectiveness, patient factors and health system factors. Patient factors were failure to attend appointments, failure to change adherence practices, and lack of consent. Health system factors were work overload, delay in getting results and drug stock outs.

**Conclusions:**

HWs are knowledgeable about the IAC program and client specific barriers should be addressed to improve viral suppression for children.

## Introduction

For a person living with HIV to benefit from anti-retroviral therapy (ART), they must be able to achieve HIV viral suppression 1. Children and adolescents living with HIV have been shown to have lower levels of viral suppression usually due to poor adherence^2^, ^3^. Psychosocial factors such as dependence on a caregiver, non-disclosure and stigma also affect viral suppression^4^–^6^. In order to help people living with HIV achieve HIV viral load suppression, the World Health Organization (WHO) recommends that heath care workers (HWs) support those with a high viral load through intensive adherence counselling (IAC)^7^. The Ministry of Health (MoH) in Uganda adopted these guidelines. They recommend the provision of three IAC sessions, one month apart, repeating the HIV viral load measurement and if it is still above 1000 copies per ml, to switch the client to a second line regimen^8^. The HWs were trained in the implementation of these guidelines/viral load algorithm and began implementation in early 2017. It was anticipated that with this intervention, 70% of clients who are non-virally suppressed, would achieve viral suppression^7^. An assessment of this intervention in 15 high volume public health sites in Uganda showed that only 77% of non-suppressed children received all three IAC sessions and 79% of these got a repeat HIV viral load test. Only 23% managed to achieve viral suppression, 41% of the non-suppressed were switched to a second line regimen and the IAC sessions took a year on average to complete and not the recommended three months^9^. There was no clear explanation from the assessment to explain these findings. The program is run principally by health workers and it was thought that they were not very conversant with the guidelines and were not knowledgeable about the viral load algorithm and this was affecting the results of the IAC program. We therefore, set out to find out the knowledge, and perceptions of health workers that support the viral load program that could explain the findings in the assessment.

## Methods

This study is part of a mixed methods study that was evaluating the viral suppression for those children and adolescents that took part in the intensive adherence program recommended by WHO in Kampala, Uganda^9^. We conducted a qualitative study with health care workers at five high volume public health facilities around Kampala district. Purposive sampling was used to invite HWs that directly oversee the clinical care-including support for adherence-of the children and adolescents to participate. Data was collected from the HWs using a semi-structured questionnaire with open-ended questions using pre-determined themes that were decided by national experts in paediatric ART adherence. Data was collected on knowledge of the viral load algorithm, and perceptions on why the program was not achieving its objectives. These in-depth interviews took place at the health facility in the ART clinic and the HWs were found at their duty stations on the day of the interview. Saturation was reached after five interviews although ten were planned. It quickly became apparent that the barriers to adherence to the viral load algorithm were client related and are reported in a different paper^10^. The interviews were conducted in English, audio recorded and transcribed verbatim. EN conducted all the data collection.

Data was analysed as it was collected. This was done manually using emergent themes that included health system, health worker, and individual factors. Four analysts reviewed all the data and determined the emerging codes from the text and questionnaires. Excerpts of the data were highlighted that spoke to the emerging codes. At the end, similar codes were grouped and emerging themes determined. The entire team agreed on the codes. One of the analysts then organized the content to help explain the observed results from the evaluation of IAC and kept returning to the data to ensure that the explanations were supported by the data. Rigor was ensured through sharing the findings with selected participants for affirmation.

## Ethical considerations

The School of Health Sciences Research Ethics Committee at Makerere University College of Health Sciences (REC No: 2017-083) and the Uganda National council of science and technology (SS 4538) provided ethical approval for this study. All the participants provided written informed consent prior to participation. To ensure confidentiality, the participants were identified by number and data was stored in a locked cabinet.

## Results

Five health care workers were interviewed, 4 (80%) were female and 1 (20%) was male. 4(80%) were nurses and 1(20%) was a clinical officer.

During assessment of the knowledge of the viral load algorithm, one of the assumptions made was that health workers did not have intimate knowledge of this algorithm. However, it quickly emerged that all the HWs were knowledgeable about the algorithm. They were also conversant with the counselling guide used for all clients with a detectable viral load. All five participants answered correctly to the structured question on the viral load algorithm.

When asked about their perceptions as to why the program seemed to be failing, two themes came up as barriers. The first was patient associated such as failure of the client to attend appointments, failure to show up for counselling sessions, representation, lack of consent and failure to implement the suggested changes that should lead to a suppressed viral load.

“*Some mothers are hesitant to switch their children to the second line regimen and they ask for more time to help the child to suppress (viral load)*”.

They indicated that if a caregiver does not bring a child to the appointment, they could not complete the sessions.

“*Some of the children are out of town with relatives in the villages; they are represented at the facility sometimes for a whole year*.”

If the caregiver fails to address the adherence issues for the child, then the viral load could not be repeated hence the low repeat viral load tests observed.

“*Sometimes caregivers return when they have not changed the way that they give the child drugs, we ask them to go back and try again*.”

The second barrier was health system related; this included delay in getting back the viral load results from the central laboratory, drug stock outs and misplaced client files on the day of appointment.

“*Delayed return of investigations such as a resistance profile can delay switching a child to second line regimen*.”

The frequent stock out of paediatric regimens also meant that a child could not be switched on that day. They would have to continue with the failing regimen until drugs became available at the health facility.

Work overload also came up as a health system barrier. They reported that they are overwhelmed with work and may not be able to accord the children and their caregivers the amount of attention required to effectively support their adherence journey.

“*At this facility, we are only two counsellors, we have to counsel the clients newly initiating ART, those who were lost to follow up and those with poor adherence. This leaves very little time for the children*.”

The work overload also meant that they did not review client records very thoroughly and often missed conducting another session.

“*Sometimes, you may not check the notes very well and you miss it (the IAC form)*.” This means that the child will have to get the next session at the next appointment, which might be two months away

Other reasons were the unfavourable school program that keeps children in boarding school for three months and possible treatment failure that could not be confirmed since drug resistance testing was reserved only for those failing on a second line regimen.

When asked about changes they would like to see in the program to make it more effective, they suggested having more counsellors, more training in how to communicate with children, and support to caregivers to disclose to the children. They also suggested having one counsellor to handle the counselling needs of each non-suppressed child to build a relationship. They called on caregivers to actively support their children's adherence as they were better placed to support good adherence.

## Discussion

In this study, we found that health care workers are knowledgeable about the viral load algorithm and their main challenges are patient related with a smaller component that is health system related. To the best of our knowledge, this is the first qualitative study to explore the knowledge and perceptions of health care workers that support the WHO recommended intensive adherence counselling program for HIV infected children and adolescents. Therefore, we have no directly related studies to compare our findings to.

Some studies have been done on the use of lay counsellors to support adherence for clients in chronic care. A systematic review of the role of lay counsellors in South Africa found that they could improve adherence but needed training and support from the health care system to recognise their contribution^11^. The HWs in this study also requested for an increase in their number to be more effective.

The patient and individual barriers that were raised by the HWs also resonated with the barriers raised by the caregivers of the children in the participating health facilities. The caregivers talked about the effect of non-disclosure, missing appointments and failure to change practice^10^. These issues were also expressed by the health care workers.

The health workers recognised that they were limited in their ability to support adherence for the children and called on caregivers to actively support adherence. This is consistent with another study done in Swaziland that showed that children and adolescents were unlikely to suppress even with adherence counselling^12^.

## Limitations

The study included only HWs in the urban district of Kampala and might not represent the views of HWs that support children and adolescents in the rural areas. The authors could not find any other qualitative study featuring HWs that support the IAC program to compare findings to.

## Conclusion

Whilst significant barriers to the intensive adherence program still exist, most are client specific and should be addressed to improve viral suppression. However, HWs' workload needs to be reduced to increase attention paid to the children. They also need extra support to become more competent in working with HIV positive children. HIV programs that implement the IAC program should train more counsellors and increase the number of counsellors that support the IAC program.
